# Predictors of mortality among under-five children in rural Ethiopia: a cross sectional study

**DOI:** 10.1186/s12887-023-04440-0

**Published:** 2023-12-15

**Authors:** Gebru Gebremeskel Gebrerufael, Bsrat Tesfay Hagos

**Affiliations:** 1 Department of Statistics, College of Natural and Computational Science, Adigrat University, P.O. Box 50, Adigrat, Ethiopia; 2 Department of Statistics, College of Natural and Computational Science, Mekelle University, P.O. Box.231, Mekelle, Ethiopia

**Keywords:** Under-five mortality, Associations, Ethiopia, Cox PH regression, EMDHS data

## Abstract

**Background:**

Under-five child mortality (UFCM) is one of the major significant and sensitive indicators of the health status of the public. Although the world has seen a remarkable and substantial decrease in UFCM since 1990, its progression rate still remains alarmingly high in Sub-Saharan African (SSA) countries, particularly in Ethiopia. Therefore, this study aimed to assess associations between mortality and under-five children in rural Ethiopia.

**Methods:**

This study used a secondary data analysis of the 2019 Ethiopia Mini Demographic and Health Survey (EMDHS) report. A total of 4,425 under-five children were included in the final analysis. The Kaplan-Meier (K-M) and Cox proportional hazard (PH) model analyses were utilized to estimate survival time and investigate the major predictors of mortality in under-five children, respectively. An adjusted hazard ratio (AHR) along with a 95% confidence interval (CI) was employed to measure the association size and direction of the association (STATA 12).

**Results:**

The study showed that 6.2% (95% CI: 5.43, 6.86) of children died beforehand celebrating their fifth birthday in rural Ethiopia. The multivariable Cox PH regression model analysis revealed associations of large spacing preceding birth interval (16–26 months) (AHR = 0.61; 95% CI: (0.402–0.920)), 27–38 months (AHR = 0.72; 95% CI: (0.496–1.03)), and ≥ 39 months, multiple births (AHR = 3.9; 95% CI: (2.77–5.62)), being breastfeeding (AHR = 0.13; 95% CI: (0.099–0.162)), and unvaccinated child (AHR = 11.6; 95% CI: (1.62–83.1)) were significant associations of under-five children mortality.

**Conclusions:**

In this study, the UFCM rate was present, with 6.2% in the rural areas of Ethiopia. The birth type, preceding birth interval, vaccination of the child, and breastfeeding are identified as significant associations with under-five child mortality in rural Ethiopia. Therefore, public health interventions should be given attention to multiple births, unvaccinated, and non-breastfeeding children, as well as mothers’ better encouragement to have a large spacing preceding the birth interval. Moreover, investigators should conduct continuous research on UFCM, which is imperative to provide current information and inform interventions in a timely manner.

**Supplementary Information:**

The online version contains supplementary material available at 10.1186/s12887-023-04440-0.

## Background

Under-five child mortality (UFCM) is one of the major significant and sensitive indicators of the health status of the public [1, 2]. Although the world has seen a remarkable and substantial decrease in child deaths in the past 3 decades, the improvement of under-five child mortality hasn’t been an effective progress in sub-Saharan African (SSA) countries and South Asia [3, 4]. Based on a systematic analysis of the global burden of disease study from 1970 to 2016, the global under-five-year-old mortality (UFCM) rate was found to be 4.10%, which requires major public health interventions [5].

Approximately 5.3 million under-five children have died worldwide since 2018. Of these, more than half (2.7 million) under-five child mortalities occur in SSA countries, including Ethiopia. The UFCM rate is 16 times higher in low-income countries than in high-income countries [6]. Particularly in Ethiopia, the 2019 EMDHS report revealed that UFCM was 59 deaths per 1,000 live births [7]. The UFCM was significantly related to maternal mental distress. This is due to women who suffered child loss reported a considerably high rate of suicidal ideation than women without child passing away [8].

Efforts to decrease under-five children’s deaths in any planned manner need a clear understanding of their associated factors. Evidence from different literature revealed that rural place of residence, unvaccinated child, poor wealth index, multiple birth type, preceding birth interval, use of unimproved water, delivery at home, sex of the child, birth orders, breastfeeding status, and antenatal care follow-up during pregnancy are among the predictors of under-five children mortality [9–12]. The majorities (80%) of the Ethiopian population were rural residents, and life expectancy and survival time (age) were comparatively short among rural residents [13, 14].

To address the problem, Ethiopia is intended to decrease the UFCM to ≤ 25 deaths per 1,000 total live births by 2030 [15, 16]. However, UFCM in Ethiopia remains one of the biggest and most difficult public health concerns that should be given focus [10, 16]. This purpose will not be attained unless the major risk predictors are extensively examined and aimed at. Even though different investigations have been carried out in Ethiopia concerning UFCM, all of them focused on both rural and urban populations, in spite of significant differences being documented [17, 18]. Moreover, in Ethiopia, differences exist in under-five years child death rates between regions. In the more resource-rich regions of Ethiopia (i.e., Adis Abeba, Tigray, Oromia, and Amhara regions), the under-five child mortality rate ranges from 39, 59, 79, and 85 deaths per 1,000 live births, respectively, whiles in the most impoverished and deprived regions of Ethiopia (i.e., Affar, Benshangul-Gumuaz, and Somali regions), it remains very high at 125, 98, and 94 deaths per 1,000 live births, respectively [19].

The 2019 EMDHS report indicated that under-five children in rural Ethiopia had, comparatively, more mortality occurrences than urban ones (i.e., 64 deaths per 1,000 live births in rural areas and 46 deaths per 1,000 live births in urban areas) [7]. Although the prevalence rate in rural Ethiopia is still high, there are limited pocket investigations carried out at the regional, town, and zone levels to assess the prevalence rate and associated risk predictors. Additionally, there is no study conducted at the national level that emphasized exactly the rural part of the country to indicate associated risk predictors of mortality occurrence among under-five children. Therefore, an evidence-based study is required for a child’s health improvement strategy by decreasing the mortality rate in under-five children in rural Ethiopia. So, this study was conducted to fill this gap by identifying the major associated factors of under-five child mortality in rural Ethiopia.

## Methods and materials

### Study design, setting, period, population, and data source

A secondary data analysis was conducted from March 21, 2019 to June 28, 2019. The 2019 EMDHS datasets were accessed from the DHS program after requesting them via formal registration. The study source and population were all under-five children in rural areas of Ethiopia, and all under-five children were selected from rural areas of Ethiopia, respectively. The datasets were limited to rural under-five children whose ages at mortality for the reduced and present age of a child for the living were recorded.

This survey was carried out by the Ethiopian Central Statistics Agency (ECSA), the Ethiopian Ministry of Health (EMoH), and the Federal Public Health Institute (FPHI). The source for this study was funded by the United States Agency for International Development (USAID).

Based on the 2019 EMDHS, report datasets were gathered using different questionnaires, and the dataset of child mortality and associated factors was obtained from a questionnaire of women who met the eligibility criteria (women of reproductive age 15–49 years).

### Sample size determination and sampling procedure

A total of 4,425 under-five children from the 2019 EMDHS dataset were included from nine regions and two administrative cities in Ethiopia. The 2019 EMDHS sample was stratified and selected in two stages. In the first stage, stratification was conducted by region, and then each region was stratified as urban and rural, yielding 21 sampling strata. A total of 305 enumeration areas (EAs) (212 in rural areas and 93 in urban areas) were selected independently in each stratum. A household listing operation was carried out in all of the selected EAs. In the second stage, a fixed number of 30 households per cluster were selected with an equal probability of systematic selection from the newly created household listing. Data coding and recording were done to reach the exact number of under-five children in the 2019 EMDHS. Moreover, the time of mortality for the reduced and the age of the child for the non-reduced were available with separate codes for all times with the respective type of respondent.

**Fig. 1:**
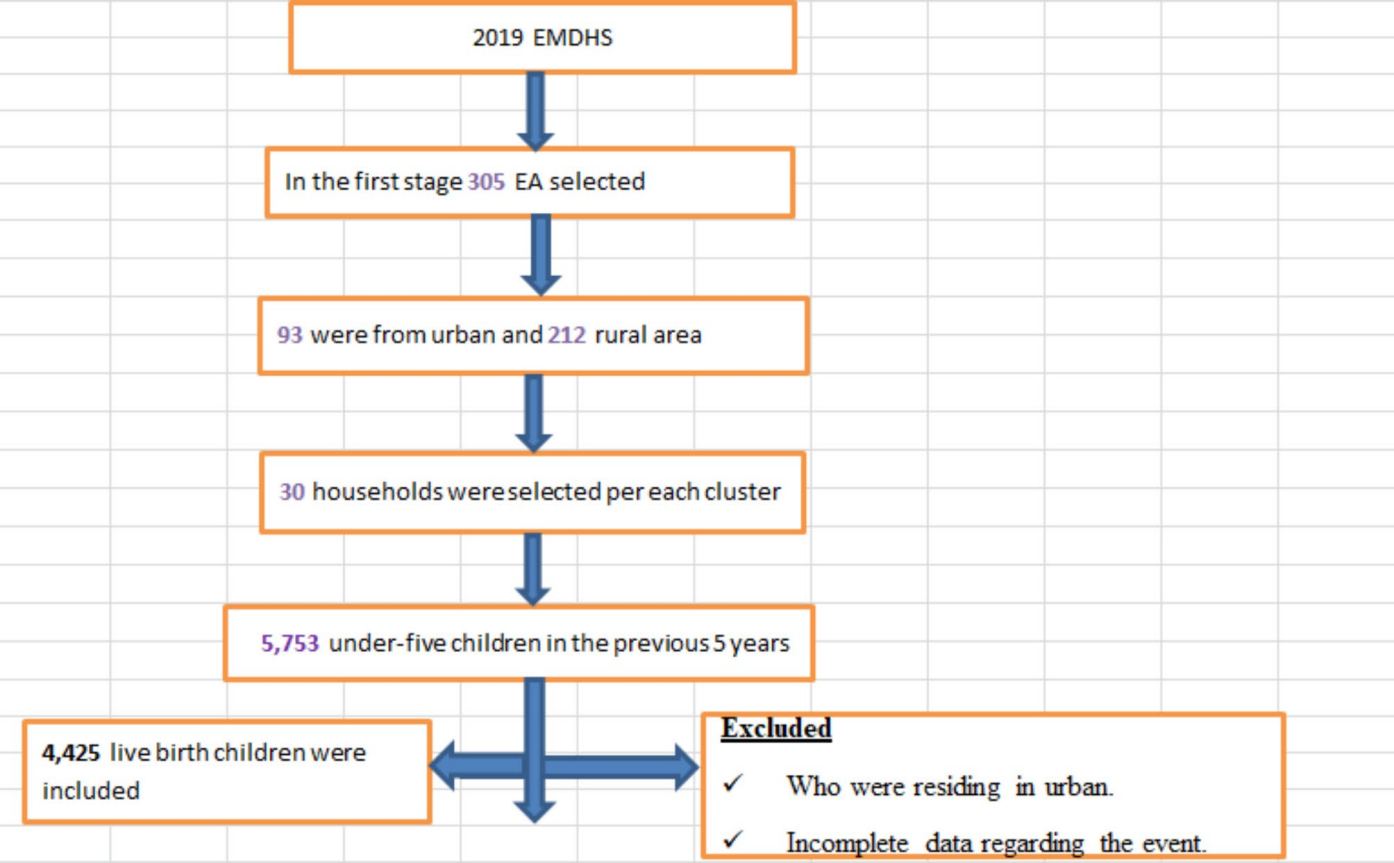
Sampling procedure of associated factors of under-five children mortality in rural Ethiopia, 2019 EMDHS

Finally, total samples of 4,425 under-five children in the 2019 EMDHS report that have completed information about all the associated factors considered were included in the study (see Fig. 1).

### Study variables

#### Response variable

The response variable (survival time-to-death) was the age of a child in months when she or he died after live birth (beginning time). Correspondingly, events (died) are defined as children who died either before or at the time of the EMDHS report dataset collection period. And whereas censored is defined as an event that did not happen (actively surviving children) until the last EMDHS data collection date [20].

#### Independent variables

According to the previously conducted studies, various socio-economic, demographic, and environmental-associated factors were included as independent variables displayed in Table 1.

**Table 1:** Operational definition and categorization of associated variables used in the study

Variables	Categorizations of independent variables
Preceding birth interval	Preceding birth interval (in month) (0 = ≤ 15, 1 = 16–26, 2 = 27–38, 3= ≥39)
Mass media access	Mass media access (0 = yes, 1 = no)
Sex of household head	Sex of household head in the family (0 = male, 1 = female)
Birth type	Birth type pregnancy (0 = singleton, 1 = multiple)
Sex of child	Sex of child (0 = male, 1 = female)
Breastfeeding	Breastfeeding (0 = no, 1 = yes)
Delivery by caesarean section	Delivery by caesarean section (0 = no, 1 = yes)
Vaccination of child	Vaccination status of child (0 = yes, 1 = no)
Maternal age	Maternal age (in years) (0 = ≤ 29, 1 = 30–34, 1 = 35–39, 2 = 40–44, 3 = 45–49)
Region	Type of regional states (1 = Tigray, 2 = Affar, 3 = Amhara, 4 = Oromia, 5 = Somali, 6 = Benshangua-gumuz, 7 = SNNPR, 8 = Gambela, 9 = Harari, 10 = Dredawa)
Education level	Educational levels of mothers (0 = higher, 1 = secondary, 2 = primary, 0 = no education)
Source of water	Source of drinking water (0 = protected, 1 = piped, 2 = unprotected)
Toilet facility	Type toilet facility (0 = yes, 1 = no )
Religion	Type of religion believes (0 = Others, 1 = Orthodox, 2 = Muslim)
Wealth index combined	Wealth index combined of households (0 = rich, 1 = middle, 2 = poor)
Birth order number	Birth order number (0 = ≤ 3, 1 = ≥ 4)
Place of delivery	Place of delivery of mothers (0 = in health facility, 1 = in home)

### Data management and analysis

The gathered data were entered into Excel in CSV format, coded, cleaned, and analyzed using the statistical software STATA version 12 (see Appendix 1). The study employed descriptive statistics like percentage distributions and frequency distributions to define the sample information. The Kaplan-Meier (K-M) non-parametric survival curve analysis was employed to compare the survival probability of predictor variables in under-five children. One of the most common types of regression models employed in survival analysis is the Cox PH model. Furthermore, this model has an important role in the investigation of the survival time-to-death of under-five children by estimating hazard ratios (HRs) and 95% confidence intervals (CIs). The general Cox PH regression model can be defined as;

1 $$\begin{array}{*{20}{l}}\begin{array}{l}{h_i}(t) = {h_0}(t)*exp({\beta _1}{{\rm{w}}_{i1}} + {\beta _2}{{\rm{w}}_{i2}} + {\beta _3}{{\rm{w}}_{i3}} + \\{\beta _4}{{\rm{w}}_{i4}} + \cdots \ldots + {\beta _P}{{\rm{w}}_{iP}})\end{array}\\\begin{array}{l}{\rm{log(}}{h_i}(t){\rm{)}} = {\rm{log}}[{h_0}(t)*exp({\beta _1}{{\rm{w}}_{i1}} + {\beta _2}{{\rm{w}}_{i2}} + \\{\beta _3}{{\rm{w}}_{i3}} + {\beta _4}{{\rm{w}}_{i4}} + \cdots \ldots + {\beta _P}{{\rm{w}}_{iP}})]\end{array}\\\begin{array}{l}= {\beta _0} + ({\beta _1}{{\rm{w}}_{i1}} + {\beta _2}{{\rm{w}}_{i2}} + {\beta _3}{{\rm{w}}_{i3}} + \\{\beta _4}{{\rm{w}}_{i4}} + \cdots \ldots + {\beta _P}{{\rm{w}}_{iP}})\end{array}\end{array}$$

This model was used to investigate and to check the impact of each independent variable on the mortality rate. Where; ${h}_{i}\left(t\right)$ is denotes the hazard for an event for patient i at time t is determined by a set of p covariates (${\text{w}}_{i1}$, ${\text{w}}_{i2}$, ${\text{w}}_{i3}$, ${\text{w}}_{i4}$……, ${\text{w}}_{ip}$), whose impact is measured by the size of the respective coefficients (${{\beta }}_{1}$, ${{\beta }}_{2}$, ${{\beta }}_{3}$, ${{\beta }}_{4}$……, ${{\beta }}_{p}$). The term ${h}_{0}\left(t\right)$ is denotes the baseline hazard for a mortality [21, 22]. The proportionality assumptions of the Cox PH regression model analysis were assured on each predictor variable and on the global test of proportionality (see Table 2).

**Table 2:** Test of proportional-hazards assumption (STATA version 12)

Predictor variables	Rho	Chisq	Df	Prob > Chisq
Preceding birth interval (ref. = ≤15)				
16–26	0.0058	0.01	1	0.924
27–38	-0.058	0.92	1	0.337
≥ 39	-0.033	0.3	1	0.58
Mass media access (ref. = yes)				
No	-0.031	0.26	1	0.608
Sex of household head (ref. = male)				
Female	-0.045	0.55	1	0.46
Birth type (ref. = single)				
Multiple	0.0278	0.25	1	0.62
Sex of child (ref. = male)				
Female	-0.032	0.29	1	0.59
Breastfeeding (ref. = no)				
Yes	0.0684	1.35	1	0.25
Delivery by caesarean section (ref. = no)				
Yes	0.036	0.37	1	0.54
Vaccination of child (ref. = yes)				
No	-0.06682	1.22	1	0.27
Global test		6.01	10	0.815

N.B:- chisq refers to chi-square statistic value, DF refers to degree of freedom, Prob > Chisq refers to *P*-value

After the PH assumption had been checked, the bivariable Cox PH regression model was fitted for each associated variable. Furthermore, those variables have a *p*-value ≤ 0.25 in the bivariable model analysis were included in the multivariable Cox PH regression model [23, 24]. Crude and adjusted hazard ratios with a 95% confidence interval (CI) were used to measure the strength the association and to explore statistically significant predictors. In the multivariable Cox PH regression model analysis, variables with a *P*-value < 0.05 were found to be significant associated factors of mortality in under-five children. The goodness of fit of the final model was checked by the Likelihood Ratio Test (LRT).

## Results

### Descriptive statistics

Overall, a total of 4,425 live births were involved in this study. Of those, 2297 (51.9%) were males and 4,289 (96.9%) were singletons. Concerning vaccination status, 3758 (84.9%) children were unvaccinated at all. The majority of 2,727 (61.6%) of children under the age of five were born in the home. Regarding children with proceeding birth intervals between (16–26 months) and (27–38 months), they encountered the highest percentage of 60 (1.4%) and 122 (2.8%) of under-five mortality, respectively. Overall, the under-five child mortality rate in rural Ethiopia was 6.2% (95% CI: 5.43, 6.86) (see Table 3).

**Table 3:** Summary of sociodemographic, economic and clinical associated factors of under-five children mortality in rural Ethiopia, from March 21, 2019, to June 28, 2019 (N = 4,425)

Variables	Categories	Censored N (%)	Death N (%)	Total
Preceding birth interval	≤ 15	263 (5.9%)	38 (0.89%)	301 (6.8%)
	16–26	841 (19%)	60 (1.4%)	901 (20.4%)
	27–38	1766 (40%)	122 (2.8%)	1888 (42.7%)
	≥ 39	1283 (29%)	52 (1.2%)	1335 (30.2%)
Mass media access	Yes	214 (4.8%)	8 (0.2%)	222 (5%)
	No	3939 (89%)	264 (6%)	4203 (95%)
Sex of household head	Male	3418 (77.2%)	232 (5.2%)	3650 (82.5%)
	Female	735 (16.6%)	40 (0.9%)	775 (17.5%)
Birth type	Single	4058 (91.7%)	231 (5.2%)	4289 (96.9%)
	Multiple	95 (2.2%)	41 (0.9%)	136 (3.1%)
Sex of child	Male	2144 (48.5%)	153 (3.5%)	2297 (51.9%)
	Female	2009 (45.4%)	119 (2.7%)	2128 (48.1%)
Breastfeeding	No	189 (4.3%)	118 (2.7%)	307 (6.9%)
	Yes	3964 (89.6%)	154 (3.5%)	4118 (93%)
Delivery by caesarean section	No	4008 (90.6%)	254 (5.7%)	4262 (96%)
	Yes	145 (3.3%)	18 (0.41%)	163 (3.7%)
Vaccination of child	Yes	666 (15.1%)	1 (0.02%)	667 (15.1%)
	No	3487 (78.8%)	271 (6.1%)	3758 (84.9%)
Maternal age	≤ 29	2369(53.5%)	151 (3.4%)	2520 (56.9%)
	30–34	887 (20.1%)	48 (1%)	935 (21.1%)
	35–39	564 (12.8%)	36 (0.8%)	600 (13.6%)
	40–44	259 (5.9%)	18 (0.4%)	277 (6.3%)
	45–49	74 (1.7%)	19 (0.4%)	93 (2.1%)
Region	Tigray	367 (8.3%)	14 (0.3%)	381 (8.6%)
	Affar	515 (11.6%)	31 (0.7%)	546 (12.3%)
	Amhara	434 (9.8%)	22 (0.5%)	456 (10.3%)
	Oromia	593 (13.4%)	39 (0.9%)	632 (14.3%)
	Somalia	491 (11%)	36 (0.8%)	527 (11.9%)
	Benshangual-gumuz	419 (9.5%)	42 (1%)	461 (10.4%)
	SNNPR	574 (13%)	27 (0.6%)	601 (13.6%)
	Gambela	325 (7.3%)	30 (0.7%)	355 (8%)
	Harari	229 (5.2%)	14 (0.3%)	243 (5.5%)
	Dredawa	206 (4.7%)	17 (0.4%)	223 (5%)
Education level	Higher	74 (1.7%)	3 (0.1%)	77 (1.7%)
	Secondary	234 (5.3%)	9 (0.2%)	243 (5.5%)
	Primary	1256 (28.4%)	97 (2.2%)	1353 (30.6%)
	No education	2589 (58.5%)	163 (3.7%)	2752 (62.2%)
Source of water	Protected water	825 (19%)	52 (1.2%)	877 (19.8%)
	Piped water	1503 (34%)	105 (2.4%)	1608 (36.3%)
	Unprotected water	1825 (41.2%)	115 (2.6%)	1940 (43.8%)
Toilet facility	Yes	337 (7.6%)	25 (0.57%)	362 (8.2%)
	No	3816 (86.2)	247 (5.6%)	4063 (91.8%)
Religion	Others	876 (20%)	52 (1.2%)	928 (21%)
	Orthodox	1110 (25.1%)	59 (1.3%)	1169 (26.4%)
	Muslim	2167 (49%)	161 (3.6%)	2328 (52.6%)
Wealth index combined	Rich	805 (18.2%)	45 (1.02%)	850 (19.2%)
	Middle	714 (16.1%)	44 (0.99%)	758 (17.1%)
	Poor	2634 (59.5%)	183 (4.1%)	2817 (63.7%)
Birth order number	≤ 3	2060 (46.6)	136 (3.1%)	2196 (49.6%)
	≥ 4	2093 (47.3%)	136 (3.1%)	2229 (50.4%)
Place of delivery	Health facility	1609 (36.4%)	89 (2%)	1698 (38.4%)
	Home	2544 (57.7%)	183 (4.1%)	2727 (61.6%)

**Fig. 2:**
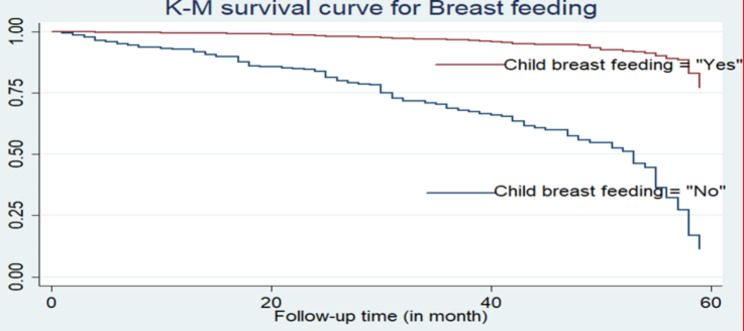
K-M survival curve for under-five children by breastfeeding

**Fig. 3:**
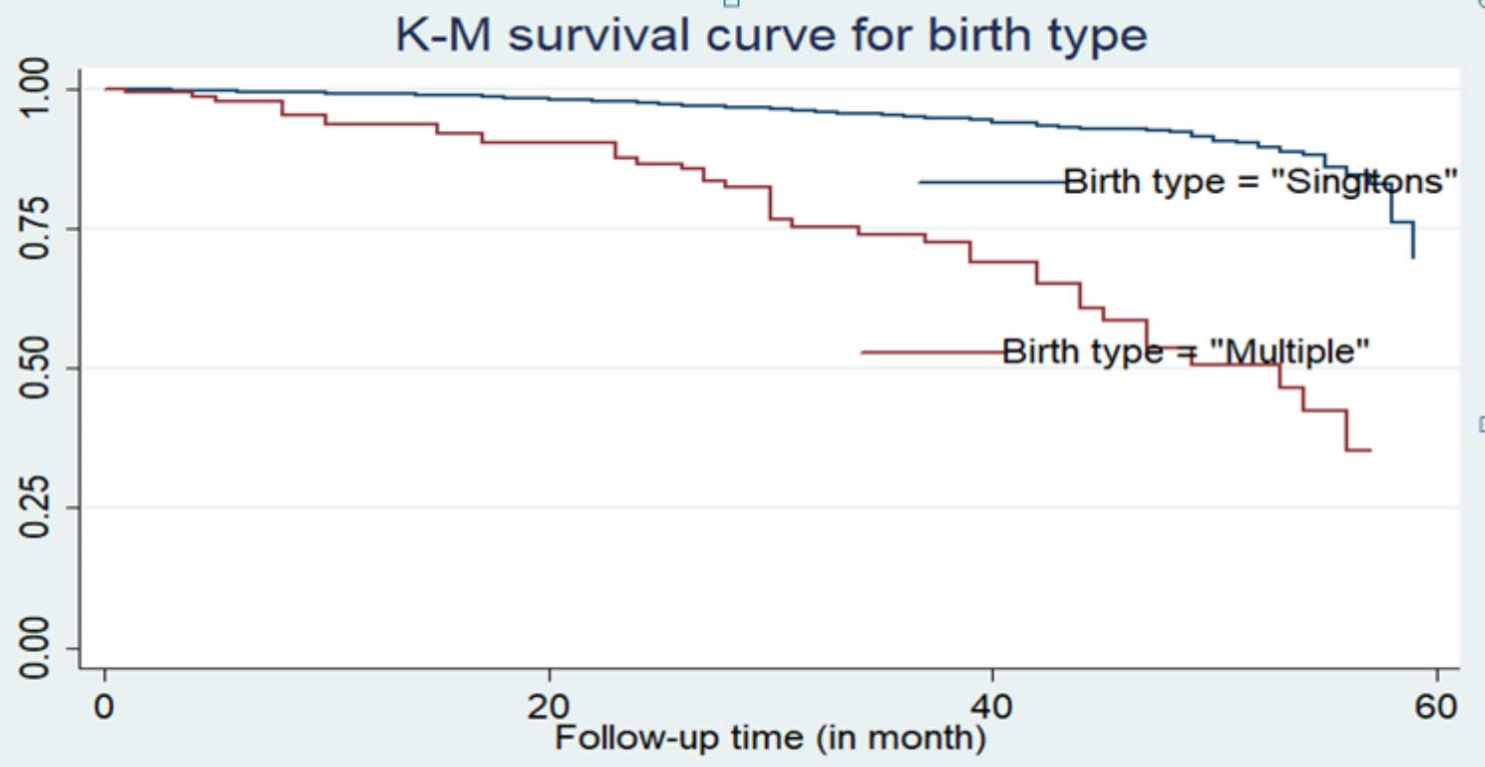
K-M survival curve for under-five children by birth type

**Fig. 4:**
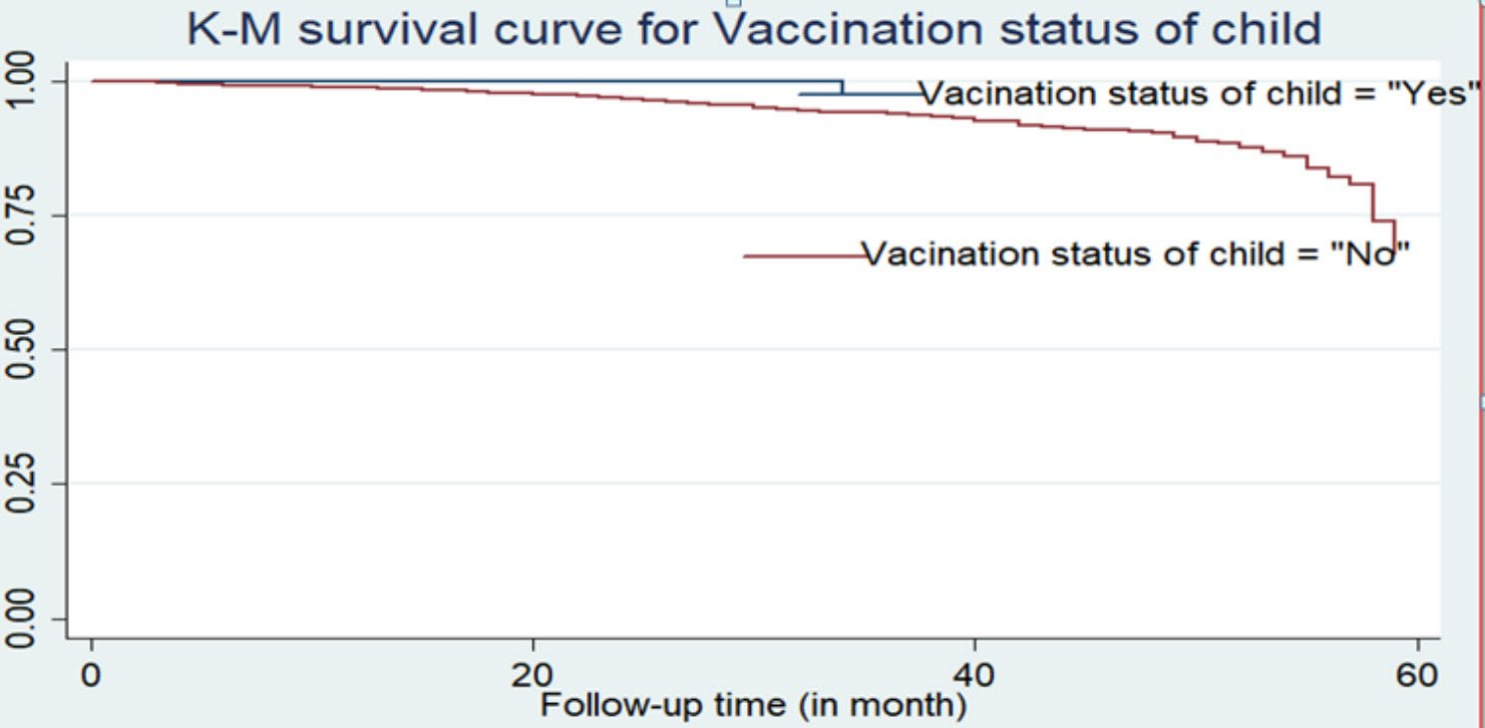
K-M survival curve for under-five children by vaccination status of child

Furthermore, according to the descriptive statistics given in Table 3, the Kaplan-Meier (K-M) non-parametric survival curve analysis estimators are plotted for the most important associated variables (see Figs. 2 and 3, and 4). These Kaplan-Meier (K-M) curves display that under-five children who do not use breastfeeding, have multiple births, and have an unvaccinated status have a short survival time compared to those reference categories.

### Associated factors of under-five children mortality in rural Ethiopia

In crude hazard ratio (CHR), the result showed that large space of preceding birth interval (16–26 months), 27–38 months, and ≥ 39 months, multiple births, being none breastfeeding, delivery by caesarean section (yes), and child vaccination (no) were significant associated factors of under-five children mortality (*P*-value < 0.05). Children who don’t vaccinate are at higher risk of mortality than vaccinated children (CHR = 18, 95% CI: 2.54–129.7). Children whose mothers were born and delivered by caesarean section (CHR = 2, 95% CI: 1.26–3.28) were at greater risk of mortality than children whose mothers were not delivered by caesarean section. Children who have multiple birth types also had a higher risk of mortality than their counterparts (CHR = 6, 95% CI: 4.46–8.72).

From the adjusted hazard ratio (AHR), the results revealed that large spacing of preceding birth interval (16–26 months), (27–38 months), and ≥ 39 months, multiple births, being non-breastfeeding, and child vaccination (no) were significant predictors of under-five children mortality (*P*-value < 0.05). The adjusted hazard ratio (AHR) of the mortality rate was 3.9 times higher among under-five children who had a multiple birth type than among those who were singleton (AHR = 3.9; 95% CI: 2.77–5.62). Similarly, when we compare the vaccination status of a child, we find it is statistically associated with the child death rate. The AHR of the child mortality rate was approximately 11.6 times higher among unvaccinated children than among vaccinated children (AHR = 11.6; 95% CI: 1.62–83.1).

There was no substantial discrepancy in the risk of child mortality rate between the sex child’s status of males and females. Furthermore, for the variable breastfeeding status of children, the AHR of the child death rate of breastfeeding children was approximately 0.13 times lower than that of children who had the none breastfeeding status of children (AHR = 0.13; 95% CI: (0.099–0.162)). The result of the likelihood ratio test (LRT) had 342.95 and a degree of freedom of 10 with a *p*-value = 0.000, which is statistically significant at the 5% level of significance, and the concordance of 0.79 explained that the dataset and the model were in good fit. Moreover, since the overall global test (*p*-value = 0.82) for all predictors is > 0.05 and none of the predictor variables failed the Cox PH model, the essential assumption (see Table 4).

**Table 4:** Bivariable and multivariable Cox PH regression model analysis of associated with under-five children mortality in Ethiopia, 2019 (N = 4,425)

Variables	CHR (95% CI)	AHR (95% CI)
Preceding birth interval (ref. = ≤15)		
16–26	0.65(0.434–0.982)	0.61(0.402–0.920)*
27–38	0.67(0.463–0.960)	0.72(0.496–1.03) *
≥ 39	0.43(0.284–0.655)	0.52(0.343–0.796) *
Mass media access (ref. = yes)		
No	1.6(0.875–3.93)	1.9 (0.877–4.00)
Sex of household head (ref. = male)		
Female	0.81(0.577–1.13)	0.82(0.581–1.14)
Birth type (ref. = single)		
Multiple	6.2( 4.46–8.72)	3.9(2.77–5.62) *
Sex of child (ref. = male)		
Female	0.84(0.661–1.07)	0.95(0.740–1.21)
Breastfeeding (ref. = no)		
Yes	0.11(0.082–0.133)	0.13(0.099–0.162) *
Delivery by caesarean section (ref. = no)		
Yes	2.0(1.26–3.28)	1.2(0.727–1.97)
Vaccination of child (ref. = yes)		
No	18( 2.54–129.7)	11.6(1.62–83.1) *

N.B:- CHR: crude hazard ratio, AHR: adjusted hazard ratio,* Significant at 5% level of significance, ref: reference, CI: confidence interval, LRT chi^2^ (10) = 342.95 and Prob > chi^2^ = 0.0000*

## Discussion

The study empirically inspected and recognized the covariates that were associated with the death rate of children in Ethiopia using the 2019 EMDHS datasets. Both descriptive statistics and multivariable Cox PH regression model analysis were used to analyze the secondary data.

In this study, 6.2% (95% CI: 5.43, 6.86) of under-five children had died before celebrating their fifth birthday, with a death rate of 62 per 1,000 live births. This finding was comparatively higher than the study conducted in Ethiopia [25] and approximately similar to the preceding 2016 Ethiopian demographic and health survey report in northern Ghana, and sub-Saharan Africa countries [19, 26, 27] but lower than other study studies done in Ethiopia [11, 12, 28, 29, 30]. The difference may be credited to variations in the study setting and sample size, as the selected sample size in our study was comparatively smaller than in others. Moreover, the difference or higher prevalence rate obtained in this study might be due to the rural residents having poor economic status, poor access to hygiene facilities, poor health care facilities, and media access for health facility usage for their new birth [31].

The multivariable Cox PH regression model analysis indicated that birth type, preceding birth interval, breastfeeding, and child vaccination status had significant associations with under-five child mortality. The hazards of under-five children’s death rate among multiple births were higher than those for singleton births. This study is concurrent with different studies done in Ethiopia [10–12, 16, 25, 26, 29]. This difference might be because multiple births can lead to growth prematurity and retardation, which are the most associated risk factors for under-five child mortality [17]. Besides, being twins may cause poor nutrition because of insufficient breast milk as well as infections due to incorrect formula breastfeeding and feeding of cow’s milk.

In the present study, the precise vaccination status of a child was an important predictor factor of the mortality rate of children in rural Ethiopia. If children were unvaccinated for a long period of time, they were more likely to die in the early newborn period compared to those who were vaccinated during the appropriate period of time. This is due to other infectious diseases, like intestinal parasites and polio. This finding is supported by previous studies done in Tigray regional states, Ethiopia [31], Ethiopia [32], and Southwest Ethiopia [33]. In such a way, health professional service workers should be given special emphasis on raising awareness among mothers about their child’s vaccination status.

Moreover, the present investigation also indicated that breastfeeding is a major associated predictor factor of under-five children’s deaths in rural Ethiopia. It was discovered that under-five children who were breastfed had decreased mortality rates among children compared to those who weren’t. This finding is consistent with the study done [25, 9, 12, 26, 34, 35]. Besides, breastfeeding has an important influence on a child’s chance of survival since the mother’s milk protects them from another infection [36, 37]. Due to this reason, children who haven’t been fed according to the suggested breastfeeding schedule are at high risk, and their survival time will be decreased. Correspondingly, short preceding birth spacing is highly associated with risk factors for under-five child mortality. This finding is in agreement with studies that indicate a significant association between the preceding birth interval and under-five child mortality [25, 10, 12, 16, 38].

### Limitations of the study

One of the limitations of this study is that EMDHS is largely based on respondents’ self-reports, which raises the possibility of recall bias given the interview’s retrospective study design and the high number of missing values in the 2019 EMDHS dataset. The other limitation of the study is that some significant predictor variables, such as the gestational mother’s age, were left out of the study. Moreover, this investigation was done four years ago, so it is unlikely to reflect the latest status of the mortality rate in the pastoral region of Ethiopia.

## Conclusion

In this study, the under-five child death rate was alarmingly high in the rural areas of Ethiopia. The birth type, preceding birth interval, vaccination of the child, and breastfeeding are identified as significant predictors of under-five child mortality in rural Ethiopia. Therefore, public health interventions should be given attention to multiple births, unvaccinated, and non-breastfeeding children, as well as mothers’ better encouragement to have a large spacing preceding the birth interval. Moreover, investigators should conduct continuous research on UFCM, which is imperative to provide current information and inform interventions timeously.

### Electronic supplementary material

Below is the link to the electronic supplementary material.

**Supplementary Material 1: Appendix 1** Syntax for Cox PH model analysis (STATA version 12)

## Data Availability

The dataset will be shared up on request and will be obtained through contacting emailing to the corresponding author (Gebru Gebremeskel Gebrerufael) using “gebrugebremeskel12@gmail.com”.

## Abbreviations

AHR: Adjusted Hazard Ratio
CHR: Crude Hazard Ratio
CI: Confidence Interval
ECSA: Ethiopian Central Statistical Agency
EMDHS: Ethiopia Mini Demographic Health Survey
K-M: Kaplan-Meier
LRT: Likelihood Ratio Test
FPHI: Federal Public Health Institute, EMoH:Ethiopian Minister of Health
PH: Proportional-Hazards
SNNPR: Southern Nations, Nationalities and People’s Region
SSA: sub-Saharan African
UFCM: Under-five Child Mortality
