# Supplementary material for: Predictors of mortality among under-five children in rural Ethiopia: a cross sectional study
Source: BMC Pediatr. 2023 Dec 15;23:633. doi: 10.1186/s12887-023-04440-0 (PMC10722689; doi:10.1186/s12887-023-04440-0)
Supplement: Supplementary file 1 — Supplementary Material 1: Appendix 1 Syntax for Cox PH model analysis (STATA version 12) [file 12887_2023_4440_MOESM1_ESM.docx]

**Appendix** 1 Syntax for Cox PH model analysis (STATA version 12)

-----------------------------------------------------------------------------------------------------------------

Cox PH model analysis

- Uni-variable Cox PH model analysis

stset Survival_time, failure (Status)
stcox i. Maternal_age i. Region i. Highest_educational_level i. Source_of_water i.Type_toilet_facility i. Mass_media_access i. Religion i. Sex_of_household_head i. Wealth_index_combined i. Birth_order_number i. Birth_type i. Sex_of_child i. Preceding_birth_interval i. Breast_feeding i. Place_of_delivery i. Vacinatin_of_child i. Delivery_by_caesarean_section

-----------------------------------------------------------------------------------------------------------------

- Multi-variable Cox PH model analysis

stset Survival_time, failure(Status)

stcox i. Mass_media_access i. Sex_of_household_head i. Birth_type i. Sex_of_child i. Preceding_birth_interval i. Breast_feeding i. Delivery_by_caesarean_section i. Vacinatin_of_child

-----------------------------------------------------------------------------------------------------------------

- Cox PH assumption

estat phtest, detail

-----------------------------------------------------------------------------------------------------------------

- Graphs of K-M curves

sts graph, by(Breast_feeding) xtitle (Follow-up time (in month)) title (K-M survival curve for Breast feeding) caption (Figure 2. Kaplan-Meier survival curve for under-five children in by Breast feeding) legend(on)

sts graph, by(Birth_type) xtitle (Follow-up time (in month)) title (K-M survival curve for birth type) caption (Figure 3. Kaplan-Meier survival curve for under-five children in by birth type) legend(on)

sts graph, by(Vacinatin_of_child) xtitle (Follow-up time (in month)) title (K-M survival curve for Vaccination status of child) caption (Figure 4. Kaplan-Meier survival curve for under-five children in by Vaccination status of child) legend(on)

-----------------------------------------------------------------------------------------------------------------
